# Health Tract, No. 29

**Published:** 1862-01

**Authors:** 


### HEALTH TRACT, No. 29.

### NEGLECTING COLDS.

(*From Hall's Journal of Health, New-York. $1 a Year.*)

EVERY intelligent physician knows that the best possible method of promptly curing a cold is, that the very day in which it is observed to have been taken, the patient should cease absolutely from eating a particle for twenty-four or forty-eight hours, and should be strictly confined to a warm room, or be covered up well in bed, taking freely hot drinks. It is also in the experience of every observant person, that when a cold is once taken, very slight causes indeed increase it. The expression, "It is nothing but a cold," conveys a practical falsity of the most pernicious character, because an experienced medical practitioner feels that it is impossible to tell in any given case, where a cold will end; hence, and when highly valuable lives are at stake, his solicitudes appear sometimes to others to verge on folly or ignorance. A striking and most instructive example of these statements is found in the case of Nicholas the First, the Emperor of all the Russias. For more than a year before his death, his confidential medical adviser observed that in consequence of the Emperor "not giving to sleep the hours needed for restoration," his general vigor was declining, and that exposures which he had often encountered with impunity, were making unfavorable impressions on the system—that he had less power of resistance. At length, while reviewing his troops on a January day, he took a severe cold, which at once excited the apprehensions of his watchful physician, who advised him not to repeat his review.

"Would you make as much of my illness if I were a common soldier?" asked the Emperor, in a tone of good-natured pleasantry.

"Certainly, please your majesty; we should not allow a common soldier to leave the hospital if he were in the state in which your majesty is."

"Well, you would do your duty—I will do mine," and the exposure was repeated, with the result of greatly increasing the bad effects of this original cold, and he died in a week afterwards.

Colds are never taken while persons are in active motion, but when at rest, just after the exercise. This result can be averted infallibly by going instantly to a warm room and remaining with all the clothing on, in which the exercise has been taken, until the body has gradually cooled down to its natural temperature, known by not feeling the slightest moisture on the forehead.

